# FOXD1 is associated with poor outcome and maintains tumor-promoting enhancer–gene programs in basal-like breast cancer

**DOI:** 10.3389/fonc.2023.1156111

**Published:** 2023-05-10

**Authors:** Kohei Kumegawa, Liying Yang, Kenichi Miyata, Reo Maruyama

**Affiliations:** ^1^ Cancer Cell Diversity Project, NEXT-Ganken Program, Japanese Foundation for Cancer Research, Tokyo, Japan; ^2^ Project for Cancer Epigenomics, Cancer Institute, Japanese Foundation for Cancer Research, Tokyo, Japan

**Keywords:** FOXD1, TCGA, gaussian mixture model, enhancer, basal-like breast cancer

## Abstract

Breast cancer biology varies markedly among patients. Basal-like breast cancer is one of the most challenging subtypes to treat because it lacks effective therapeutic targets. Despite numerous studies on potential targetable molecules in this subtype, few targets have shown promise. However, the present study revealed that *FOXD1*, a transcription factor that functions in both normal development and malignancy, is associated with poor prognosis in basal-like breast cancer. We analyzed publicly available RNA sequencing data and conducted *FOXD1*-knockdown experiments, finding that *FOXD1* maintains gene expression programs that contribute to tumor progression. We first conducted survival analysis of patients grouped via a Gaussian mixture model based on gene expression in basal-like tumors, finding that *FOXD1* is a prognostic factor specific to this subtype. Then, our RNA sequencing and chromatin immunoprecipitation sequencing experiments using the basal-like breast cancer cell lines BT549 and Hs578T with *FOXD1* knockdown revealed that *FOXD1* regulates enhancer–gene programs related to tumor progression. These findings suggest that *FOXD1* plays an important role in basal-like breast cancer progression and may represent a promising therapeutic target.

## Introduction

The biology of breast cancer, a leading cause of cancer-related death among women worldwide, varies greatly among patients (1, 2), and studies on gene expression profiles have revealed several intrinsic subtypes, e.g., luminal A, luminal B, HER2-enriched, normal-like, and basal-like (3–6). The most aggressive subtype, basal-like breast cancer, is characterized by a gene expression profile similar to that of basal or myoepithelial cells. However, basal-like breast cancer tumors often lack established therapeutic targets, such as hormone receptors or HER2, making their treatment challenging (7). Moreover, the heterogeneity of basal-like tumors makes identifying optimized therapeutic targets difficult.

In the epigenetic landscape of cancer, the complex and dynamic interplay between genetic and environmental factors has profound implications on the initiation, progression, and metastasis of malignant neoplasms (8). The aberrant regulation of cis-regulatory elements and transcription factors (TFs) is a key mechanism underlying reprogramming of the gene regulatory circuit in cancer and leads to the emergence of distinct phenotypic and functional states (9). Enhancers, a class of noncoding cis-regulatory elements, are characterized by specific histone modifications, such as acetylation of histone H3 lysine 27 (H3K27ac), which confer a permissive chromatin environment and promote the transcriptional activity of target genes. The aberrant activation of TFs, particularly those associated with oncogenic pathways, is one of the most common mechanisms underlying enhancer reprogramming in cancer (10). For example, in breast cancer, abnormal upregulation of the luminal-lineage TF *FOXA1* modifies genome-wide enhancer activity and induces transcriptional reprogramming to establish an endocrine-resistant state in metastatic tumors (11).

Previous studies have found that *FOXD1*, a member of the forkhead TF family, plays a critical role in regulating various cellular and molecular processes in both normal and malignant tissues (12–16). The expression of *FOXD1* is upregulated in primary breast cancer and promotes tumor proliferation and chemoresistance in the MDA-MB-231 (basal-like) and MCF7 (luminal) cell lines (17). However, the specific effects of *FOXD1* in basal-like tumors, mechanisms underlying these effects, and association with enhancer–gene regulation have not been elucidated.

In the present study, we conducted integrative analysis of publicly available expression data and multiomics data from cell line experiments to identify potential therapeutic targets for basal-like breast cancer. We stratified patients with basal-like tumors in The Cancer Genome Atlas Breast Invasive Carcinoma (TCGA-BRCA) data using a Gaussian mixture model (GMM) according to the expression level of each gene. We then performed survival analysis to identify genes associated with poor prognosis in basal-like breast cancer, finding that *FOXD1* was associated with poor prognosis in basal-like breast cancer but not in other subtypes. RNA sequencing (RNA-seq) and H3K27ac chromatin immunoprecipitation sequencing (ChIP-seq) experiments in basal-like cell lines with *FOXD1* knockdown revealed that *FOXD1* maintains distinct enhancer-gene programs associated with tumor progression. Collectively, our findings suggest that *FOXD1* plays a critical role in establishing an aggressive phenotype in a basal-like breast cancer subset by maintaining tumor-promoting epigenetic features and gene expression patterns.

## Materials and methods

### Quantification of FOXD1 expression in breast cancer cell lines

Breast cancer cell lines were either purchased from the Japanese Collection of Research Bioresources (JCRB) or American Type Culture Collection (ATCC) or were kindly gifted by Drs. Hitoshi Zembutsu and Yoshio Miki. The cells were maintained in accordance with the manufacturer’s instructions, and details were provided in **Supplementary Table 1**. Total RNA was extracted from the cells using QIAGEN RNeasy Plus Mini Kit (QIAGEN). Subsequently, cDNA was synthesized from 500 ng of total RNA using PrimeScript RT Master Mix Perfect Realtime (TaKaRa) and then diluted to 200 µL. Reverse transcription real-time polymerase chain reaction (RT–qPCR) was performed using 2 µL of cDNA per reaction with PowerUp™ SYBR™ Green Master Mix (ThermoFisher) via 7500 Fast Real-Time PCR System (Applied Biosystems). The relative expression levels of *FOXD1* were determined using the delta-delta Ct method, with normal human breast tissue RNA (BioChain) as a reference, and the endogenous housekeeping gene ACTB serving as an internal control. The primers used in this study included FOXD1-F (GGACTCTGCACCAAGGGA), FOXD1-R(AAACACCGAACCACCAAGAC), ACTB-F(GCCAACCGCGAGAAGATGA), and ACTB-R(AGCACAGCCTGGATAGCAAC).

### Small interfering RNA transfection

BT549 and Hs578T cells were seeded onto 6-well plates at a density of 1.6 × 10^5^ cells per well and cultured for 24 h. Cells were transfected with FOXD1 small interfering RNA (siRNA) (Ambion, s5229 or s5230) and negative siRNA control (Ambion, Negative Control siRNA) at a final concentration of 16 nM using Lipofectamine RNAiMAX Transfection Reagent (Invitrogen) and Opti-MEM Reduced Serum Medium (Gibco) following the manufacturer’s instructions. Cells were subjected to RNA-seq and ChIP-seq 48 h after transfection. Knockdown efficiency was confirmed by quantifying FOXD1 mRNA as described above.

### RNA-seq

Total RNA was extracted from BT549 and Hs578T cells that had been treated with siRNA for 48 h, using the method described previously. RNA-seq libraries were prepared with 10 ng of total RNA, as a technical duplicate, using a SMARTer Stranded Total RNA-Seq Kit v2-Pico Input Mammalian (Takara) following the manufacturer’s instructions. The resulting gene expression libraries were sequenced via an Illumina NextSeq 550 platform with paired-end reads (read1, 75 bp; index1 8 bp; read2, 75 bp).

### ChIP-seq

BT549 and Hs578T cells treated with siRNA for 48 h were subjected to ChIP-seq. ChIP-seq was performed as described in our previous report with slight modifications (18). Briefly, approximately 3 × 10^5^ cells were fixed with 0.5% formaldehyde for 10 min at room temperature, quenched with 1.25 M glycine, washed, and lysed. The chromatin was sheared using an S220 Focused-ultrasonicator (Covaris). The complex of anti-H3K27ac antibody (Cell Signaling Technology, 8173S) and protein G Dynabeads (Invitrogen) was mixed with sheared chromatin. After overnight incubation, the complex was washed and incubated at 65°C for 4 h for reverse cross-linking. The released DNA was purified using AMPure XP (Beckman). ChIP-seq libraries were prepared as a technical duplicate using ThruPLEX DNA-seq Kit (Takara) and were sequenced via Illumina NextSeq.

### Cell growth assay

BT549 and Hs578T cells were seeded onto 96-well plates at a density of 3 × 10^3^ cells per well. After 24 h, siRNA was transfected into the cells using the same method and concentration as described previously. After 5 h, RealTime-Glo™ MT Cell Viability Assay (Promega) reagent was added to the cells, and luminescence was quantified at 48 h using ARVO2 (PerkinElmer).

### The cancer genome atlas breast invasive carcinoma data analysis

We downloaded TCGA-BRCA RNA-seq data as a SummarizedExperiment object via the R package TCGAbiolinks (19) using “GDCquery(project = “TCGA-BRCA,” data.category = “Transcriptome Profiling,” data.type = “Gene Expression Quantification,” workflow.type = “STAR–Counts”),” “GDCdownload(),” and “GDCprepare().” GMM clustering was performed using the “Mclust()” function in the mclust package with the option modelNames = “V.” For survival analysis, we used the “survfit()” function of the survival package and “ggsurvplot()” function of the survminer package.

### RNA-seq analysis

To generate an expression count matrix, row reads were trimmed to remove adaptor sequences using Skewer (v0.2.2) and mapped to the hg38 genome using STAR (v.2.7.8a). The mapped reads were then counted using featureCounts (v.2.0.10). To quantify differential gene expression, we utilized edgeR’s glmQLFTest (v3.32.1). We used control and intervention experiments as input groups with a simple design using a 0 intercept ‘~0 + Group.’ First, we normalized the library sizes by calculating scaling factors using ‘calcNormFactors(y, method = TMM).’ We next estimated dispersions via ‘estimateDisp(y, design = design, robust = TRUE)’ and fitted the generalized linear model using ‘glmQLFit(y, design = design).’ Finally, we determined log2 fold changes and false discovery rates (FDRs) using glmQLFTest. Genes with an FDR of <0.01 and log2FC of >1 (upregulated) or <−1 (downregulated) were considered differentially expressed genes. Gene Ontology enrichment analysis was performed via Enrichr (20).

### ChIP-seq analysis

The sequenced reads were mapped to the hg38 genome using bowtie2 (v.2.4.2). Multi-mapped reads and PCR duplicates were removed using Picard (v.2.25.3). Overlapping reads according to the ENCODE blacklist were filtered out using bedtools (2.30.0). MACS2 (v.2.2.7.1) was used for calling peaks with the “–keep-dup auto -q 0.1” parameter. After calling peaks, the peak summits were extended by 250 bp on both sides to a final width of 501 bp. Then, the regions of ENCODE hg38 blacklist were filtered out. Overlapping peaks within a single sample were removed using an iterative removal procedure that preserved the most significant peaks based on MACS2 ‘score’ values, thus identifying “a sample peak set.” The “score per million” was calculated by dividing individual peak score by the sum of all peak scores in each sample divided by 1 million. This iterative removal procedure was repeated across sample peak sets based on the score per million. The reproducible peak set was identified by selecting peaks with a score per million of ≥5 and overlaps between at least two samples; in addition, the peaks on chromosome Y were removed. Finally, we generated a reproducible high-quality set of 501 bp fixed-width peaks. To obtain the number of fragments in each peak, bam files were read as Genomic Ranges object via R using Rsamtool’s “scambam().” Each fragment per peak was estimated using “countOverlaps().” The counts matrix was normalized using edgeR’s “cpm(log = TRUE, prior.count = 1).” The motif enrichment score was calculated using ChromVAR (21) as follows: (i) adding GC bias information using “addGCBias(),” (ii) identifying elements with motifs via “matchMotifs()” using the motif annotation of the R package chromVARmotif’s “homer_pwms,” (iii) obtaining background peaks via “getBackgroundPeaks(),”and (iv) calculating motif deviations using “computeDeviations().” The Z-scores of motif deviations (i.e., Motif scores) were used for analysis. To perform differential peak analysis, we used edgeR as previously described for RNA-seq analysis. Peaks with an FDR of <0.1 and log2FC of >0 (upregulated) or <0 (downregulated) were determined as differential peaks. To annotate peaks and determine peak–gene association, Genomic Regions Enrichment of Annotations Tool (GREAT) was used with default settings.

## Results

### GMM based approach reveals genes associated with overall survival in basal-like breast cancer

**Figure 1:**
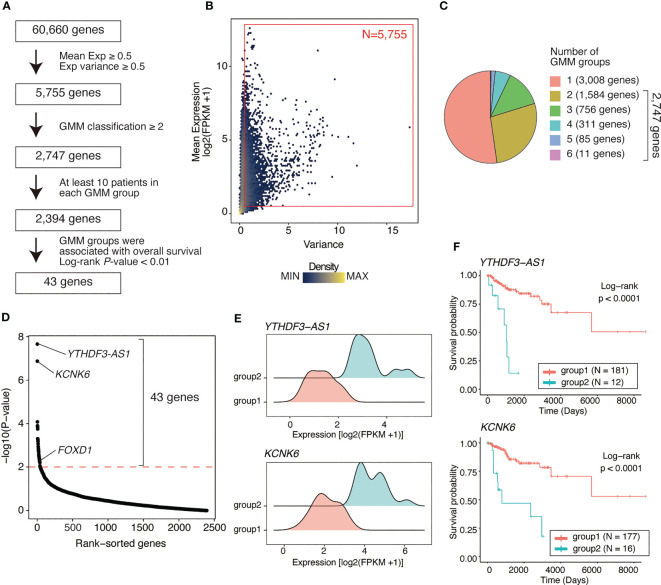
Identifying genes associated with poor outcome in basal-like breast cancer using a Gaussian mixture model. **(A)** Flow chart of gene identification. First, genes with low mean expression or low variance (both mean expression [log2(FPKM+1)] and variance of <0.5) across basal-like breast cancer samples were filtered out, leaving 5755 genes. Using GMM classification, 2747 genes that divided the patients into ≥2 expression groups were selected. Thus, based on gene expression, 2394 genes covering ≥2 GMM groups containing ≥10 patients were selected. Finally, 43 were identified as the genes whose expression level were associated with poor prognosis (Kaplan–Meier analysis; log-rank P-value of <0.01). **(B)** Scatter plot showing mean expression level and variance of expression of each gene. Each dot represents a gene. Red box represents the selected genes (N = 5755, average expression ≥ 0.5; variance ≥ 0.5). **(C)** Pie chart showing the distribution of the number of GMM groups. Overall, 2747 genes were selected covering ≥2 expression level-based GMM groups. **(D)** Dot plot showing log-rank test *P*-values of each filtered gene (2,394 genes) calculated via Kaplan–Meier analysis between GMM clusters. Red line represents filtering criteria (*P* < 0.01), and 43 genes were finally selected as the prognostic factors in basal-like breast cancer. **(E)** Ridge plots representing the distribution of *YTHDF3-AS1* and *KCNK6* expression in GMM groups. **(F)** Kaplan–Meier plots of patient groups stratified by the GMM for *YTHDF3-AS1* and *KCNK6* expression*. P*-values were calculated using log-rank test.

RNA-seq data from 193 basal-like tumors in the TCGA-BRCA dataset were analyzed to identify prognosis-associated genes in basal-like breast cancer. Genes were screened using three steps: (1) filtering genes with low average expression and variance of expression, (2) classifying tumors based on the expression level of each gene using the GMM, and (3) comparing the survival rates between different GMM classifications via Kaplan–Meier analysis (**Figure 1A**).

Of 60,660 genes, 5,755 genes had both an average expression and variance of expression of ≥0.5 across basal-like tumors (**Figure 1B**). Using GMM classification, we stratified the patients into one group (not stratified) for 3,008 genes, two groups for 1,584 genes, three groups for 756 genes, four groups for 311 genes, five groups for 85 genes, and six groups for 11 genes (**Figure 1C**). For 2,394 genes with ≥2 GMM groups that contained at least 10 patients per group, we conducted survival analysis, identifying 43 genes for which expression groups were associated with overall survival (log-rank P-value <0.01; **Figure 1D** and **Supplementary Table 2**). For example, *YTHDF3-AS1* and *KCNK6* were the top significant genes associated with poor prognosis. According to their expression levels, the patients were stratified into two groups, i.e., the low and high expression groups (**Figure 1E**), of which the high expression group exhibited shorter overall survival (**Figure 1F**). Although *YTHDF3-AS1* has not been well-characterized, its antisense gene, *YTHDF3*, is involved in the progression and metastasis of triple-negative tumors (22). *KCNK6* is as an overexpressed gene that promotes breast cancer cell proliferation, invasion, and migration (23). Overall, these results indicate that the GMM-based approach is useful for identifying genes associated with patient outcomes.

### *FOXD1* expression is associated with poor outcome in basal-like breast cancer but not in other subtypes

**Figure 2:**
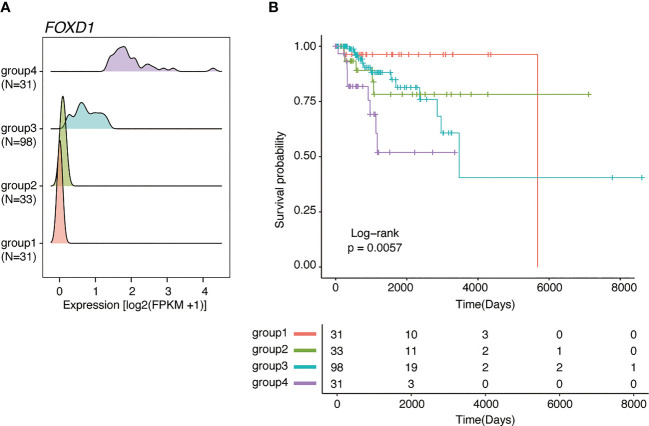
*FOXD1* is associated with poor prognosis specifically in basal-like breast cancer. **(A)** Ridge plots representing the distribution of *FOXD1* expression for GMM groups (group1, N=31; group2, N=33; group3, N=98; group4, N = 31). **(B)** Kaplan–Meier plot of *FOXD1* expression-based GMM groups stratified by GMM for *FOXD1* expression*. P*-values were calculated using log-rank test.

Of the 43 prognosis-related genes identified, we focused on *FOXD1*, a TF involved in breast cancer proliferation and drug resistance. According to *FOXD1* expression levels, the GMM classified patients into four groups (**Figure 2A**), of which the groups with higher *FOXD1* expression levels (groups 3 and 4) exhibited poorer outcomes (**Figure 2B**). According to GMM classification by *FOXD1* expression and survival analysis in the other subtypes, there was no significant difference across the GMM classification for luminal A, luminal B, Her2-enriched, and normal-like tumors (**Supplementary Figure 1**). These results suggest that elevated *FOXD1* expression is associated with poor outcome specifically in basal-like breast cancer.

### *FOXD1* regulates distinct gene expression in basal-like breast cancer cell lines

**Figure 3:**
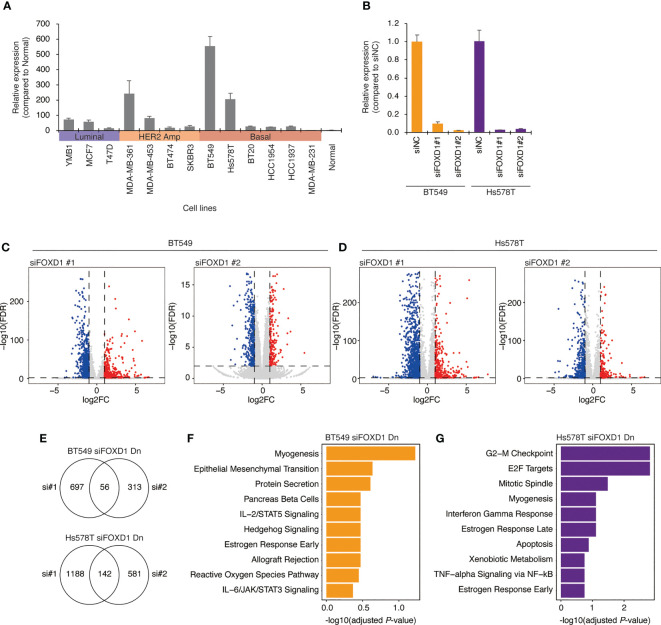
Gene expression changes induced by *FOXD1*-KD in basal-like breast cancer cell lines BT549 and Hs578T. **(A)** Bar chart showing *FOXD1* expression levels in breast cancer cell lines (three luminal, four HER2 amplification, six basal-like lines) and normal breast tissue examined *via* qPCR. Error bars represent standard deviation. **(B)** Bar chart showing *FOXD1* expression levels in BT549 and Hs578T cells transfected with control siRNA (siNC) or two siRNA against FOXD1 (siFOXD1#1 and siFOXD1#2). Error bars represent standard deviation. **(C)** Volcano plot showing differential expression analysis in BT549 cells between siNC and siFOXD1#1 (left) or siFOXD1#2 (right). Each dot represents a gene. The upregulated or downregulated genes are indicated in red or blue, respectively. **(D)** Volcano plot showing differential expression analysis in Hs578T cells between siNC and siFOXD1#1 (left) or siFOXD1#2 (right). Each dot represents a gene. The upregulated or downregulated genes are indicated in red or blue, respectively. **(E)** Venn diagram showing overlaps between the genes downregulated by siFOXD1#1 and siFOXD1#2 in BT549 (up) or Hs578T (bottom) cells. **(F, G)** Bar chart showing gene enrichment analysis (MSigDB Hallmark gene set) for the overlap of the downregulated genes by two siRNAs in BT549 **(F)** and Hs578T **(G)** cells.

To confirm our findings, we examined *FOXD1* expression levels in a panel of breast cancer cell lines, including three luminal lines (MCF7, T47D, and YMB1), four HER2-amplified lines (BT474, SKBR3, MDA-MB-361, and MDA-MB-453), and six basal lines (BT549, Hs578T, MDA-MB-231, BT20, HCC1954, and HCT1937). We found that *FOXD1* expression levels were low in all luminal lines, with high expression levels observed only in one HER2-amplified line (MDA-MB-361) and two basal cell lines (BT549 and HS578T) (**Figure 3A**). These results were consistent with the heterogeneity of *FOXD1* expression found among basal-like tumors in our analysis of TCGA-BRCA data (**Figure 2A**).

To investigate the impact of *FOXD1* on gene expression, we conducted *FOXD1*-knockdown (*FOXD1*-KD) experiments using siRNA in BT549 and Hs578T cells, followed by RNA-seq analysis. First, we validated the effectiveness of two different siRNAs against FOXD1 (siFOXD1#1 and siFOXD1 #2) (**Figure 3B**). Subsequently, we performed RNA-seq analysis and identified 56 genes in BT549 cells and 142 genes in Hs578T cells that were commonly downregulated by *FOXD1*-KD using two siRNAs (**Figures 3C–E** and **Supplementary Table 3**). We performed gene annotation analysis using The Molecular Signature Database Hallmark gene set and found that the genes downregulated by *FOXD1*-KD in BT549 cells were associated with myogenesis, and those in Hs578T cells were associated with the G2–M checkpoint (**Figures 3F, G**), suggesting that FOXD1 has a different effect on the gene expression program of each cell line. Interestingly, cell proliferation assays following *FOXD1*-KD did not indicate significant inhibition of cell growth in either cell line (**Supplementary Figure 2**). This suggests that although FOXD1 plays a role in tumor progression and shorter prognosis, it may not be involved in cell cycle regulation.

### *FOXD1* regulates enhancer–gene programs potentially associated with tumor progression

Enhancers play a critical role in maintaining gene expression programs in various tumors. To examine the impact of FOXD1 on enhancer activity, we conducted *FOXD1*-KD in BT549 and Hs578T cells, followed by H3K27ac ChIP-seq assays. Our analysis identified 25,669 and 53,794 consensus peaks in BT549 and Hs578T cells, respectively. Using principal component analysis, we observed distinct patterns of enhancer activity in both cells treated with control siRNA (siNC), siFOXD1#1, and siFOXD1#2 (**Supplementary Figure 3**).

**Figure 4:**
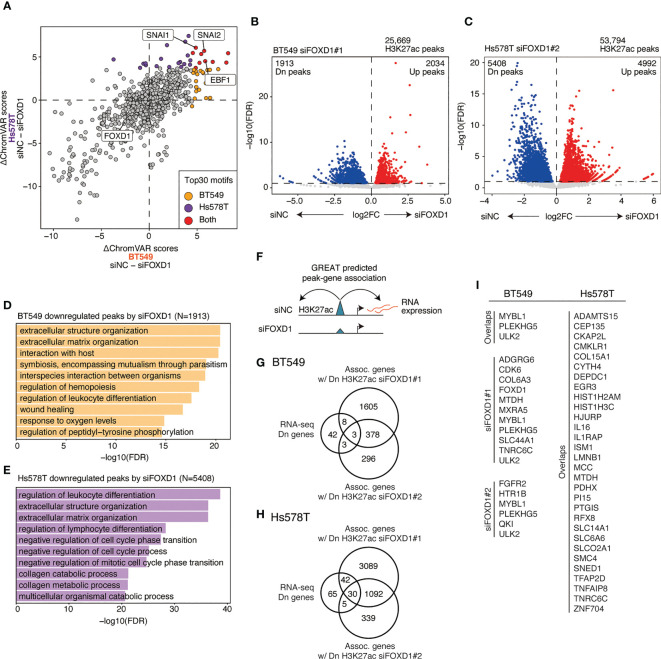
Changes in enhancer activity induced by *FOXD1*-KD and enhancer–gene pairs maintained by FOXD1. **(A)** Scatter plot of ΔChromVAR motif scores (siNC − siFOXD1) calculated using H3K27ac ChIP-seq data. A high ΔChromVAR score indicates reduced motif enrichment in H3K27ac peaks by *FOXD1*-KD. Each dot represents a CISBP motif. The top 30 enriched motifs in each cell line are indicated in orange (BT549), purple (Hs578T), or red (both). **(B)** Volcano plot showing differential peak analysis in BT549 cells between siNC and siFOXD1#1. Each dot represents a peak. Upregulated or downregulated peaks are indicated in red or blue, respectively. **(C)** Volcano plot showing differential peak analysis in Hs578T cells between siNC and siFOXD1#1. Each dot represents a peak. Upregulated or downregulated peaks are indicated in red or blue, respectively. **(D)** Bar plot showing the GREAT GO Biological Process for H3K27ac peaks downregulated by siFOXD1#1 in BT549 cells. **(E)** Bar plot showing the GREAT GO Biological Process for H3K27ac peaks downregulated by siFOXD1#1 in Hs578T cells. **(F)** Schema representing the identification for downregulated peak–gene associations via *FOXD1*-KD. **(G)** Venn diagram showing overlaps between the genes downregulated by *FOXD1*-KD and the genes associated with the downregulated H3K27ac peaks by siFOXD1#1 and siFOXD1#2 in BT549 cells. **(H)** Venn diagram showing overlaps between the genes downregulated by *FOXD1*-KD and the genes associated with the downregulated H3K27ac peaks by siFOXD1#1 and siFOXD1#2 in Hs578T cells. **(I)** List of enhancer–gene pairs. For Hs578T cells, only overlapping genes are included (other pairs are shown in **Supplementary Table 6**).

To identify TFs that regulate FOXD1-associated enhancers, we used ChromVAR to calculate TF motif scores and found that EBF1, SNAI1, and SNAI2 were less enriched in both cell lines after *FOXD1*-KD (**Figure 4A** and **Supplementary Table 4**). EBF1 is a tumor-promoting TF in triple-negative breast cancer (24), whereas SNAI1/2 are master regulatory TFs for organogenesis and wound healing in normal tissue as well as for the epithelial–mesenchymal transition (EMT) in cancer cells (25). Interestingly, enhancers containing FOXD1 motifs themselves were not downregulated, indicating an indirect effect of FOXD1 for maintaining enhancer activity. These results suggest that FOXD1 is involved in maintaining the activity of the enhancers targeted by tumor-promoting TFs.

Next, we performed differential analysis of the control and *FOXD1*-KD groups, where siFOXD1#1 and siFOXD1#2 identified 1,913 and 485 downregulated peaks in BT549 cells (**Figure 4B** and **Supplementary Figure 4A**) and 5,408 and 1,133 downregulated peaks in Hs578T cells, respectively (**Figure 4C** and **Supplementary Figure 4B**). GREAT Gene Ontology analysis revealed that these regions were associated with tumor microenvironment features such as extracellular structure organization, hemopoiesis regulation, and blood vessel morphogenesis (**Figures 4D, E** and **Supplementary Figures 4C, D**). In BT549 cells, the downregulated peaks were also associated with wound healing (**Figure 1D**), which was consistent with the lower SNAI1/2 motif enrichment in *FOXD1*-KD (**Figure 4A**).

To identify enhancer–gene pairs that could be regulated by FOXD1, we identified genes that were transcriptionally downregulated and were associated with the downregulated peaks in *FOXD1*-KD identified via GREAT analysis (**Figure 4F**). We found that 14 and 82 enhancer–gene pairs were downregulated by *FOXD1*-KD (at least one of the siRNAs) in BT549 or Hs578T cells, respectively (**Figures 4G, H**; **Supplementary Tables 5, 6**). Interestingly, we observed that these pairs were largely distinct between the two cell lines (**Figure 4I**; **Supplementary Tables 5 and 6**), indicating that the function of FOXD1 depends on the context. Both gene lists contained genes associated with tumor progression and metastasis, such as *CDK6*, *SNED1*, and *MTDH (* 26–28). Taken together, these findings suggest that *FOXD1* modulates gene expression programs involved in tumor progression.

## Discussion

Forkhead box TFs are involved in various processes of cancer progression such as metastasis, hormone regulation, therapeutic resistance, and reprogramming metabolism (29). FOXA1 is a central regulator of gene expression programs in ER+ breast cancer (30), FOXC1 is associated with EMT and poor prognosis in basal-like breast cancer (31), and FOXO3 is often dysregulated and plays both tumor-suppressive and oncogenic roles (32, 33). FOXD1 plays a role in both normal development and cancer progression. In normal development, FOXD1 controls kidney morphogenesis (34, 35) and appropriate formation of the optic chiasm (36). FOXD1 also contributes to the successful reprogramming of cells during the establishment of induced pluripotent stem cells (37). Moreover, it has been suggested that FOXD1 plays critical roles in cell proliferation, invasion, metastasis, and poor prognosis in various cancer types. In glioma, FOXD1 has been found to activate signaling pathways that contribute to the tumorigenicity of mesenchymal glioma stem cells by activating *ALDH1A3* transcription (38). A recent report also suggested that FOXD1 enhances GLUT1 expression, leading to cell proliferation, invasion, and metastasis by modulating aerobic glycolysis in pancreatic cancer (15). Similarly, FOXD1 regulates histone modification to promote tumor growth in clear cell renal cell carcinoma (16). These results suggest that FOXD1 is involved in tumor progression and is a promising target for multiple cancer types.

In this study, we analyzed publicly available expression data from clinical specimens, finding that elevated expression of *FOXD1* is associated with poor outcomes in basal-like breast cancer but not in other subtypes. Therefore, FOXD1 may be a lineage-specific tumor-promoting TF in basal-like breast cancer. We also analyzed gene expression and enhancer profiles in the BT549 and Hs578T cell lines, finding that some enhancer-genes were regulated by FOXD1. The binding motifs of EMT-associated TFs SNAI1/2 were enriched in the potential enhancers regulated by FOXD1, suggesting that FOXD1 can modulate the enhancer activity regulating dedifferentiation. These enhancer–gene pairs included *CDK6*, [a key regulator of cell cycle and other tumor-promoting programs (26)], *SNED1* [a metastasis-promoting gene associated with poor prognosis of triple-negative breast cancer (27)], and MTDH [a gene involved in breast cancer initiation, metastasis, and drug resistance (28)]. These findings are consistent with those of previous reports regarding FOXD1 function associated with tumor progression and metastasis. Further, they highlight that FOXD1 may be an oncogenic TF that activates tumor-promoting gene expression programs by modulating enhancers. Although FOXD1-KD downregulated cancer-associated enhancer–gene pairs, we did not observe any effect on the proliferation of either of the cell lines (**Supplementary Figure 2**). This result may seem contradictory to the RNA-seq results in Hs578T cells, which showed that *FOXD1*-KD downregulated the expression of cell cycle-associated genes (**Figure 3G**). However, we also observed that enhancers downregulated by *FOXD1*-KD were associated with EMT-related TFs (**Figure 4A**), suggesting that FOXD1 is more closely related to metastatic features than to cell proliferation.

The present study and other studies suggest that targeting FOXD1 is an attractive therapeutic approach for basal-like breast cancer. Accordingly, inhibiting FOXD1 could potentially reduce breast cancer metastasis by inactivating the enhancers associated with EMT. Furthermore, recent studies have shown that molecular targeting therapies can increase the sensitivity of established treatments, such as chemotherapy and radiotherapy, in various types of cancer (39–41). One of the primary challenges in developing FOXD1-targeting therapies is the lack of available FOXD1 inhibitors. TFs are generally not considered druggable, but recent progress in developing proteolysis-targeting chimera (PROTAC) technology has made it possible to target certain TFs. For example, a clinical trial for ARV-471, an estrogen receptor (ER) degrader, demonstrated significant clinical efficacy in patients with ER-positive breast cancer (42). This implicates that FOXD1 specific inhibitors could be developed using PROTAC technology. Therefore, further research is warranted to develop specific FOXD1 inhibitors and determine the precise role of FOXD1 in cancer using breast cancer preclinical models.

This study has a limitation of using only cell line models for investigating FOXD1 function. Although cell lines are useful tools for cancer biology, they may exhibit distinct features compared with primary tumors. To address this limitation, further research should focus on manipulating patient-derived models, such as patient-derived organoids and xenografts. Despite this limitation, our study is clinically relevant because it reveals the correlation between poor prognosis and *FOXD1* expression levels in basal-like primary tumors and indicates that FOXD1 maintains specific enhancer–gene programs associated with tumor progression.

In summary, we used integrative analysis of TCGA-BRCA RNA-seq data and cell line experiments to highlight the intertumor heterogeneity of gene expression in basal-like tumors and identify a gene set associated with poor prognosis in basal-like breast cancer. *FOXD1* knockdown experiments revealed that FOXD1 maintains the regulation of enhancers associated with tumor-promoting gene expression in basal-like cell lines. Based on our findings, we postulate that FOXD1 is a critical TF that influences the epigenetic machinery underlying tumor progression and may be a potential therapeutic target.

## Data availability statement

Sequencing data have been deposited at GEO (GSE230119) and are publicly available as of the date of publication. R code for reproducing the analysis is available at https://github.com/KoheiKumegawa/BasalBC_GMM.

## Author contributions

KK and KM performed data analysis. LY performed cell line and sequencing experiments RM supervised all the work. KK and RM wrote the manuscript. All authors contributed to the article and approved the submitted version.

## References

[1] DeSantis CE Ma J Gaudet MM Newman LA Miller KD Goding Sauer A. Breast cancer statistics, 2019. CA Cancer J Clin (2019) 69:438–51. doi: 10.3322/caac.21583 31577379

[2] Sung H Ferlay J Siegel RL Laversanne M Soerjomataram I Jemal A. Global cancer statistics 2020: GLOBOCAN estimates of incidence and mortality worldwide for 36 cancers in 185 countries. CA Cancer J Clin (2021) 71:209–49. doi: 10.3322/caac.21660 33538338

[3] Sotiriou C Pusztai L. Gene-expression signatures in breast cancer. New Engl J Med (2009) 360:790–800. doi: 10.1056/NEJMra0801289 19228622

[4] Sørlie T Tibshirani R Parker J Hastie T Marron JS Nobel A. Repeated observation of breast tumor subtypes in independent gene expression data sets. Proc Natl Acad Sci (2003) 100:8418–23. doi: 10.1073/pnas.0932692100 PMC166244 12829800

[5] Sørlie T Perou CM Tibshirani R Aas T Geisler S Johnsen H. Gene expression patterns of breast carcinomas distinguish tumor subclasses with clinical implications. Proc Natl Acad Sci (2001) 98:10869–74. doi: 10.1073/pnas.191367098 PMC58566 11553815

[6] Perou CM Sørlie T Eisen MB van de Rijn M Jeffrey SS Rees CA. Molecular portraits of human breast tumours. Nature (2000) 406:747–52. doi: 10.1038/35021093 10963602

[7] Rakha EA Reis-Filho JS Ellis IO. Basal-like breast cancer: a critical review. J Clin Oncol (2008) 26:2568–81. doi: 10.1200/JCO.2007.13.1748 18487574

[8] Hanahan D. Hallmarks of cancer: new dimensions. Cancer Discovery (2022) 12:31–46. doi: 10.1158/2159-8290.CD-21-1059 35022204

[9] Sur I Taipale J. The role of enhancers in cancer. Nat Rev Cancer (2016) 16:483–93. doi: 10.1038/nrc.2016.62 27364481

[10] Okabe A Kaneda A. Transcriptional dysregulation by aberrant enhancer activation and rewiring in cancer. Cancer Sci (2021) 112:2081–8. doi: 10.1111/cas.14884 PMC8177786 33728716

[11] Fu X Pereira R de Angelis C Veeraraghavan J Nanda S Qin L. FOXA1 upregulation promotes enhancer and transcriptional reprogramming in endocrine-resistant breast cancer. Proc Natl Acad Sci (2019) 116:26823–34. doi: 10.1073/pnas.1911584116 PMC6936436 31826955

[12] Kobayashi A Mugford JW Krautzberger AM Naiman N Liao J McMahon AP. Identification of a multipotent self-renewing stromal progenitor population during mammalian kidney organogenesis. Stem Cell Rep (2014) 3:650–62. doi: 10.1016/j.stemcr.2014.08.008 PMC4223698 25358792

[13] Hernández-Bejarano M Gestri G Monfries C Tucker L Dragomir EI Bianco IH. Foxd1-dependent induction of a temporal retinal character is required for visual function. Development (2022) 149. doi: 10.1242/dev.200938 PMC9845753 36520654

[14] Sun Q Novak D Hüser L Poelchen J Wu H Granados K. FOXD1 promotes dedifferentiation and targeted therapy resistance in melanoma by regulating the expression of connective tissue growth factor. Int J Cancer (2021) 149:657–74. doi: 10.1002/ijc.33591 33837564

[15] Cai K Chen S Zhu C Li L Yu C He Z. FOXD1 facilitates pancreatic cancer cell proliferation, invasion, and metastasis by regulating GLUT1-mediated aerobic glycolysis. Cell Death Dis (2022) 13:765. doi: 10.1038/s41419-022-05213-w 36057597 PMC9440910

[16] Bond KH Fetting JL Lary CW Emery IF Oxburgh L. FOXD1 regulates cell division in clear cell renal cell carcinoma. BMC Cancer (2021) 21:312. doi: 10.1186/s12885-021-07957-8 33761914 PMC7988646

[17] Zhao Y-F Zhao J-Y Yue H Hu K-S Shen H Guo Z-G. FOXD1 promotes breast cancer proliferation and chemotherapeutic drug resistance by targeting p27. Biochem Biophys Res Commun (2015) 456:232–7. doi: 10.1016/j.bbrc.2014.11.064 25462566

[18] Maruyama R Choudhury S Kowalczyk A Bessarabova M Beresford-Smith B Conway T. Epigenetic regulation of cell type–specific expression patterns in the human mammary epithelium. PloS Genet (2011) 7:e1001369. doi: 10.1371/journal.pgen.1001369 21533021 PMC3080862

[19] Colaprico A Silva TC Olsen C Garofano L Cava C Garolini D. TCGAbiolinks: an R/Bioconductor package for integrative analysis of TCGA data. Nucleic Acids Res (2016) 44(8):e71. doi: 10.1093/nar/gkv1507 26704973 PMC4856967

[20] Xie Z Bailey A Kuleshov MV Clarke DJB Evangelista JE Jenkins SL. Gene set knowledge discovery with enrichr. Curr Protoc (2021) 1. doi: 10.1002/cpz1.90 PMC8152575 33780170

[21] Schep AN Wu B Buenrostro JD Greenleaf WJ. ChromVAR: inferring transcription-factor-associated accessibility from single-cell epigenomic data. Nat Methods (2017) 14:975–8. doi: 10.1038/nmeth.4401 PMC5623146 28825706

[22] Lin Y Jin X Nie Q Chen M Guo W Chen L. YTHDF3 facilitates triple-negative breast cancer progression and metastasis by stabilizing ZEB1 mRNA in an m6A-dependent manner. Ann Transl Med (2022) 10:83–3. doi: 10.21037/atm-21-6857 PMC8848410 35282088

[23] Hou X Tang L Li X Xiong F Mo Y Jiang X. Potassium channel protein KCNK6 promotes breast cancer cell proliferation, invasion, and migration. Front Cell Dev Biol (2021) 9:616784. doi: 10.3389/fcell.2021.616784 34195184 PMC8237943

[24] Qiu Z Guo W Dong B Wang Y Deng P Wang C. EBF1 promotes triple-negative breast cancer progression by surveillance of the HIF1α pathway. Proc Natl Acad Sci (2022) 119(28):e2119518119. doi: 10.1073/pnas.2119518119 35867755 PMC9282371

[25] Georgakopoulos-Soares I Chartoumpekis DV Kyriazopoulou V Zaravinos A. EMT factors and metabolic pathways in cancer. Front Oncol (2020) 10:499. doi: 10.3389/fonc.2020.00499 32318352 PMC7154126

[26] Fassl A Geng Y Sicinski P. CDK4 and CDK6 kinases: from basic science to cancer therapy. Sci (1979) (2022) 375:eabc1495. doi: 10.1126/science.abc1495 PMC9048628 35025636

[27] Naba A Clauser KR Lamar JM Carr SA Hynes RO. Extracellular matrix signatures of human mammary carcinoma identify novel metastasis promoters. Elife (2014) 3:e01308. doi: 10.7554/eLife.01308 24618895 PMC3944437

[28] Shen M Wei Y Kim H Wan L Jiang Y-Z Hang X. Small-molecule inhibitors that disrupt the MTDH–SND1 complex suppress breast cancer progression and metastasis. Nat Cancer (2021) 3:43–59. doi: 10.1038/s43018-021-00279-5 35121987 PMC8818087

[29] Castaneda M den Hollander P Mani SA. Forkhead box transcription factors: double-edged swords in cancer. Cancer Res (2022) 82:2057–65. doi: 10.1158/0008-5472.CAN-21-3371 PMC9258984 35315926

[30] Seachrist DD Anstine LJ Keri RA. FOXA1: a pioneer of nuclear receptor action in breast cancer. Cancers (Basel) (2021) 13:5205. doi: 10.3390/cancers13205205 34680352 PMC8533709

[31] Han B Bhowmick N Qu Y Chung S Giuliano AE Cui X. FOXC1: an emerging marker and therapeutic target for cancer. Oncogene (2017) 36:3957–63. doi: 10.1038/onc.2017.48 PMC5652000 28288141

[32] Yao S Fan LY-N Lam EW-F. The FOXO3-FOXM1 axis: a key cancer drug target and a modulator of cancer drug resistance. Semin Cancer Biol (2018) 50:77–89. doi: 10.1016/j.semcancer.2017.11.018 29180117 PMC6565931

[33] Khan MA Ahmad I Aloliqi AA Eisa AA Najm MZ Habib M. FOXO3 gene hypermethylation and its marked downregulation in breast cancer cases: a study on female patients. Front Oncol (2023) 12:1078051. doi: 10.3389/fonc.2022.1078051 36727057 PMC9885168

[34] Hatini V Huh SO Herzlinger D Soares VC Lai E. Essential role of stromal mesenchyme in kidney morphogenesis revealed by targeted disruption of winged helix transcription factor BF-2. Genes Dev (1996) 10:1467–78. doi: 10.1101/gad.10.12.1467 8666231

[35] Levinson RS Batourina E Choi C Vorontchikhina M Kitajewski J Mendelsohn CL. Foxd1-dependent signals control cellularity in the renal capsule, a structure required for normal renal development. Development (2005) 132:529–39. doi: 10.1242/dev.01604 15634693

[36] Herrera E Marcus R Li S Williams SE Erskine L Lai E. Foxd1 is required for proper formation of the optic chiasm. Development (2004) 131:5727–39. doi: 10.1242/dev.01431 15509772

[37] Koga M Matsuda M Kawamura T Sogo T Shigeno A Nishida E. Foxd1 is a mediator and indicator of the cell reprogramming process. Nat Commun (2014) 5:3197. doi: 10.1038/ncomms4197 24496101

[38] Cheng P Wang J Waghmare I Sartini S Coviello V Zhang Z. FOXD1–ALDH1A3 signaling is a determinant for the self-renewal and tumorigenicity of mesenchymal glioma stem cells. Cancer Res (2016) 76:7219–30. doi: 10.1158/0008-5472.CAN-15-2860 PMC5161538 27569208

[39] Pawar JS Al-Amin M Hu C-D. JNJ-64619178 radiosensitizes and suppresses fractionated ionizing radiation-induced neuroendocrine differentiation (NED) in prostate cancer. Front Oncol (2023) 13:1126482. doi: 10.3389/fonc.2023.1126482 36959798 PMC10028149

[40] Owens JL Beketova E Liu S Shen Q Pawar JS Asberry AM. Targeting protein arginine methyltransferase 5 suppresses radiation-induced neuroendocrine differentiation and sensitizes prostate cancer cells to radiation. Mol Cancer Ther (2022) 21:448–59. doi: 10.1158/1535-7163.MCT-21-0103 PMC8898292 35027481

[41] Shu S Wu HJ Ge JY Zeid R Harris IS Jovanović B. Synthetic lethal and resistance interactions with BET bromodomain inhibitors in triple-negative breast cancer. Mol Cell (2020) 78:1096–1113.e8. doi: 10.1016/j.molcel.2020.04.027 32416067 PMC7306005

[42] Békés M Langley DR Crews CM. PROTAC targeted protein degraders: the past is prologue. Nat Rev Drug Discovery (2022) 21:181–200. doi: 10.1038/s41573-021-00371-6 35042991 PMC8765495

