# Supplementary material for: FOXD1 is associated with poor outcome and maintains tumor-promoting enhancer–gene programs in basal-like breast cancer
Source: Front Oncol. 2023 May 10;13:1156111. doi: 10.3389/fonc.2023.1156111 (PMC10206236; doi:10.3389/fonc.2023.1156111)
Supplement: Supplementary file 1 [file DataSheet_1.pdf]

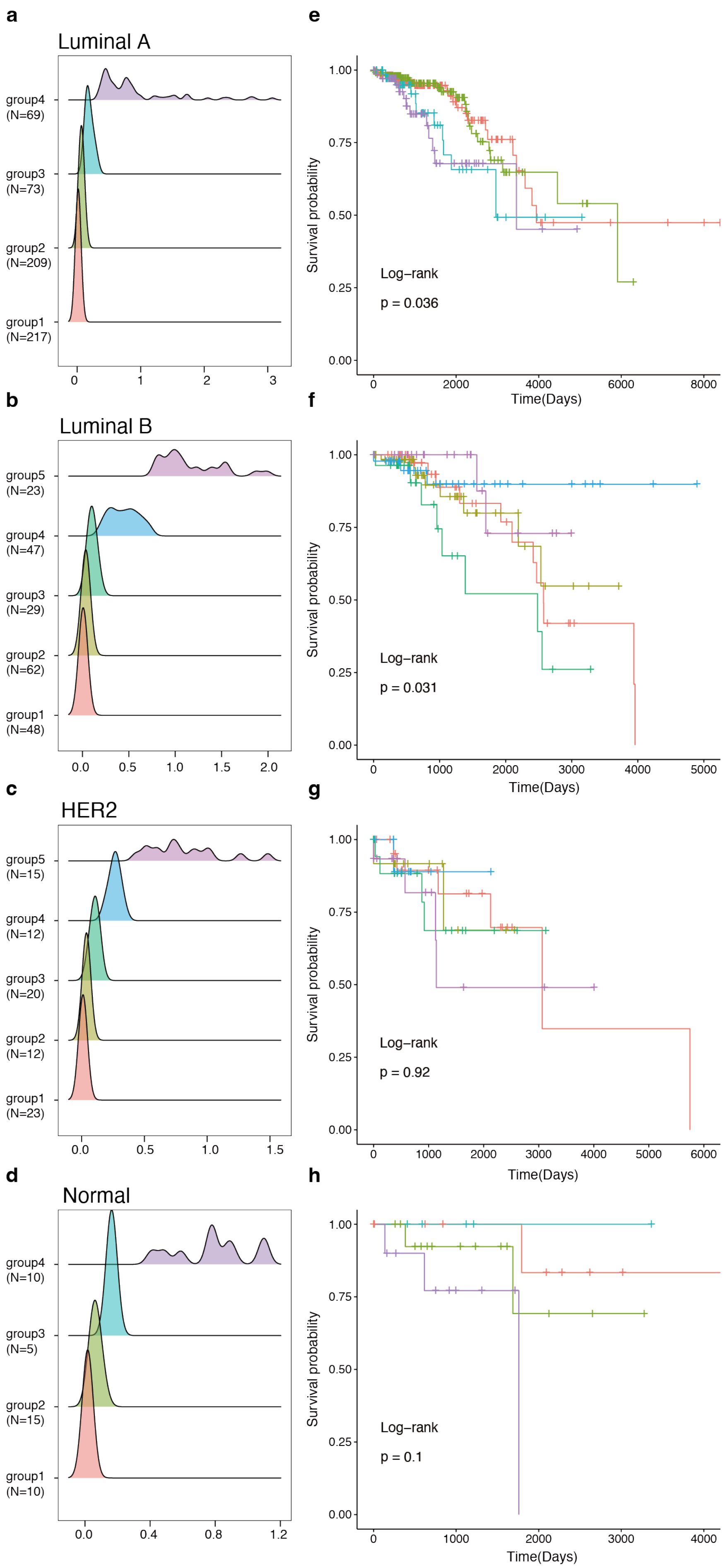

Supplementary Figure 1. (a–d) Ridge plot representing the distribution of FOXD1 expression for GMM groups of luminal A (a), luminal B (b), HER2 (c), and normal-like (d). (e–h) Kaplan-Meier plot for the patient groups stratified by GMM groups of luminal A (e), luminal B (f), HER2 (g), and normal-like (h).

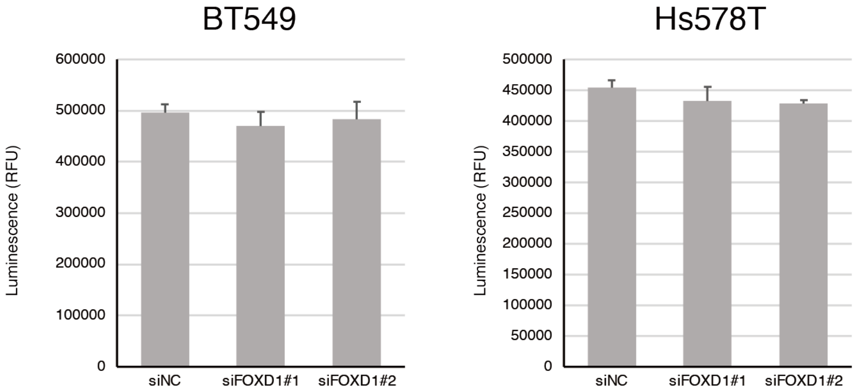

Supplementary Figure 2. Cell proliferation assay by FOXD1-KD. Bar plot showing the luminescence 48 hrs after siRNA transfection in BT549 (left) and Hs578T (right)

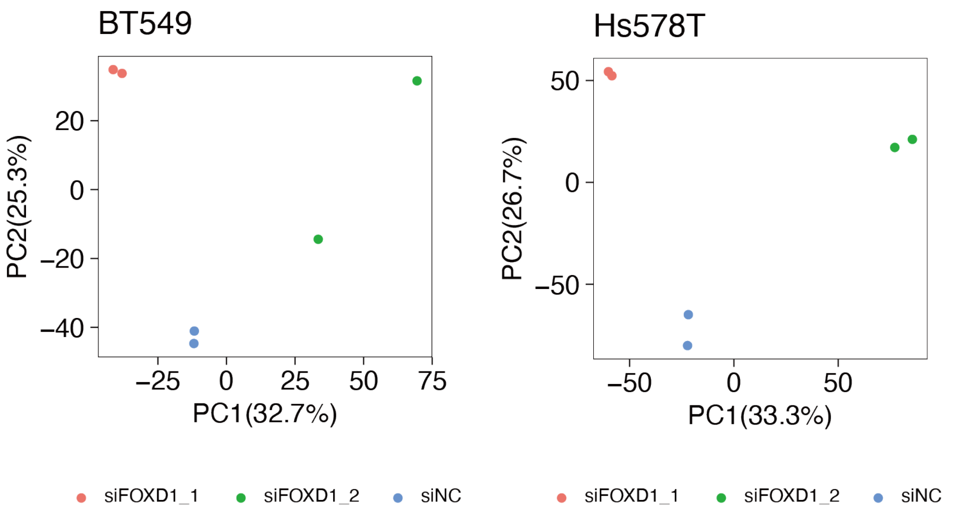

Supplementary Figure 3. Principal component analysis of H3K27ac ChIP-seq with siRNA treatment in BT549 and Hs578T

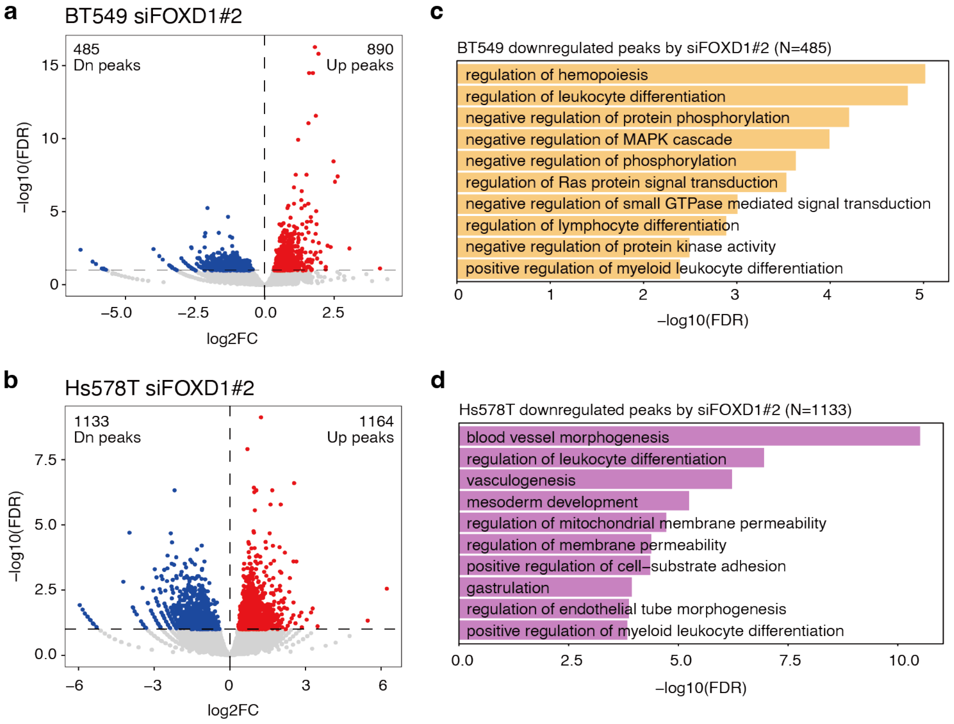

Supplementary Figure 4. Enhancer activity changes by FOXD1-KD. (a) Volcano plot showing differential peak analysis in BT549 between siNC and siFOXD1#2. Each dot represents each peak. The upregulated or downregulated peaks are red-colored or blue-colored. (b) Volcano plot showing differential peak analysis in Hs578T between siNC and siFOXD1#2. Each dot represents each peak. The upregulated or downregulated peaks are red-colored or blue-colored. (c) Bar plot showing GREAT GO Biological Process for the downregulated H3K27ac peaks by siFOXD1#2 in BT549. (d) Bar plot showing GREAT GO Biological Process for the downregulated H3K27ac peaks by siFOXD1#1 in Hs578T.
